# Supplementary material for: Systematic analysis of exonic germline and postzygotic de novo mutations in bipolar disorder
Source: Nat Commun. 2021 Jun 18;12:3750. doi: 10.1038/s41467-021-23453-w (PMC8213845; doi:10.1038/s41467-021-23453-w)
Supplement: Supplementary file 1 — Supplementary Information [file 41467_2021_23453_MOESM1_ESM.pdf]

Table of Contents

Supplementary Note 1 .... **2**

Consideration on the origin of pzDNMs in *SRCAP* 2

Supplementary Tables **4**

Supplementary Table 1. Data obtained from previous studies or databases 4

Supplementary Table 2. Sample information and sequencing platforms for *de novo* mutation (DNM) analysis 5

Supplementary Table 3. Workflow of two pipelines for gDNM detection 6

Supplementary Table 4. Computational annotation for the detected variants 7

Supplementary Table 5. Workflow for pzDNM detection 8

Supplementary Table 6. CHIP candidate genes in previous studies 9

Supplementary Figures **10**

Supplementary Figure 1. An overview of the study design 10

Supplementary Figure 2. Enrichment of LoF gDNMs hitting constrained genes in the group of BD excluding SCZAD 11

Supplementary Figure 3. Detailed information on marker gene expression in cell cluster analysis 12

Supplementary Figure 4. Properties of the validated pzDNMs in BD 13

Supplementary Figure 5. Preprocessing for the single-cell (nucleus) enrichment analysis of the genes hit by deleterious gDNMs in BD 14

Supplementary Note 1

Consideration on the origin of pzDNMs in *SRCAP*

Given relatively high VAF of the pzDNMs detected from whole-exome sequencing data of saliva or blood DNA in our study, the majority of these are likely pzDNMs of early developmental origin shared by multiple tissues including the brain. However, there is another possibility that these pzDNMs are derived from age-related clonal hematopoiesis (note that the major source of saliva DNA is white blood cells). In the case of LoF pzDNM in *KMT2C* (p.Lys3601*), we could determine that this variant is a pzDNM of early developmental origin by examining multiple tissues. However, in the cases of two BD probands carrying a deleterious pzDNM in *SRCAP*, we could not obtain other tissues. We therefore carefully evaluated the possibility that the deleterious pzDNMs in *SRCAP* are derived from age-related clonal hematopoiesis having no critical role in brain disorders by reviewing literature and analyzing the data of pzDNMs in non-psychiatric individuals.

First, we investigated if *SRCAP* is included in the list of 87 candidate genes implicated in age-related clonal hematopoiesis of indeterminate potential (CHIP) from six previous studies[101-106] (**Supplementary Table 6**) or the list of 576 established oncogenic genes (TCGA576, https://portal.gdc.cancer.gov/). We verified that *SRCAP* is not in either of the two lists, indicating that there is no strong evidence that deleterious pzDNMs in *SRCAP* confer a proliferative advantage. Second, we scrutinized the list of pzDNMs in 4,528 unaffected parents of ASD probands in the study by Krupp et al.[107] (**Supplementary Table 1**). After reannotating the pzDNMs in the Krupp et al. study with the same procedure as our analysis of pzDNMs in BD, there are 771 high-confidence (PHET_MAIN < 0.001 in their study) deleterious pzDNMs in 4,528 unaffected parents (average age = 41.0). Of these, there is one damaging missense pzDNM in *SRCAP*. Based on the mutation rate for each gene provided by Samocha et al.[39], this observed proportion (one in 771) is not significantly different from the theoretical expectation (0.000331447) calculated from the mutation rate of deleterious DNMs in *SRCAP* (0.00009588445) and that of deleterious DNMs in all protein-coding genes (0.289290674) with an available mutation rate (18,271 genes) ($P = 0.232$ without multiple testing correction, one-tailed binomial test). Therefore, *SRCAP* is not a gene frequently mutated in blood-derived DNA samples from individuals with similar ages as the two BD probands carrying a deleterious *SRCAP* pzDNM (44 and 46 years of age at the recruitment). Third, a whole-exome sequencing study of blood-derived DNA samples from 17,182 individuals who were not selected for hematologic phenotypes showed that pzDNMs are detected only in a small fraction of the individuals with 40 to 49 years of

age (~1.7%), whereas such mutations are more frequent in persons older than 70 years of age (~10.1%)[104]. Given the above-described ages of the BD probands with a deleterious *SRCAP* pzDNM, the probability that the pzDNMs in these individuals are derived from age-related clonal hematopoiesis should not be high. Taken these together, we conclude that there is no evidence supporting that the deleterious *SRCAP* pzDNMs observed in BD are derived from age-related clonal hematopoiesis.

Supplementary Table 1. Data obtained from previous studies or databases

Item	Source	File
Theoretical mutation rate	Samocha et al. Nat Genet. 2014	Supplementary Table 1
DD gene	Kaplanis et al. Biorxiv 2019	Supplementary Table2
BD GWAS index gene	Stahl et al. Nat Genet. 2019	Table 1
Proportion of damaging missense DNMs in control	Satterstrom et al. Cell 2020	Supplementary Table 1
DNM list in schizophrenia	Fromer et al. Nature 2014	Supplementary Table 1[a]
DNM list in schizophrenia	Howrigan et al. Nat Neurosci. 2020	Supplementary Data 4
DNM list in schizophrenia	Rees et al. Nat Neurosci. 2020	Supplementary Table 12[b]
DNM list in ASD and control	Satterstrom et al. Cell 2020	Supplementary Table 1
DNM list in DD	Kaplanis et al. Biorxiv 2019	Supplemental Table 1
DNM list in *KMT2C*	Kleefstra et al. Am J Hum Genet. 2012	Table 3
DNM list in *KMT2C*	Koemans et al. PLoS Genet. 2017	Table 1
DNM list in *KMT2C*	Faundes et al. Am J Hum Genet. 2018	Table 1
SRCAP protein domain	Hood et al. Am J Hum Genet. 2012	Figure 2
pzDNM list in ASD parents	Krupp et al. Am J Hum Genet. 2017	Supplementary Table S5
PantherGOslim (GO term list)	http://pantherdb.org/panther/goSlim.jsp	PantherGOslim.obo
Gene list from GO consortium	http://current.geneontology.org/products/pages/downloads.html	goa.human.gaf
Gene list from SynGO	https://www.syngoportal.org/index.html	syngo_ontologies.xlsx
Gene list from EnricherGO	http://amp.pharm.mssm.edu/Enrichr/#stats	GO_Biological_Process_2018, GO_Cellular_Component_2018, GO_Molecular_Function_2018
Gene list for EnrichmentMap	http://download.baderlab.org/EM_Genesets	Human_GO_AllPathways_no_GO_iea_March_01_2020_symbol.gmt
GTEx v8	https://gtexportal.org/home/datasets	GTEx_Analysis_2017-06-05_v8_RNASeQCv1.1.9_gene_median_tpm.gct.gz
BrainSpan	http://genetics.wustl.edu/jdlab/psi_package/	pSI.data_1.0.tar > data/human.rda
Single-nucleus RNA sequencing data of human ACC	https://portal.brain-map.org/atlases-and-data/rnaseq	human_ACC_2018-10-04_exon-matrix.csv
KMT2C protein structure	https://www.ncbi.nlm.nih.gov/gene/58508	Gene ID: 58508
Multiz Alignments of 100 Vertebrates track	https://genome.ucsc.edu/	UCSC genome browser

[a]Excluding 86 probands with SCZAD. [b]Several probands with SCZAD are included, but cannot be distinguished from the list in the publication.

Supplementary Table 2. Sample information and sequencing platforms for *de novo* mutation (DNM) analysis

Diagnosis	Data source	N of trios	Tissue	Exon capture	Sequencer
Bipolar disorder (BD)	Our recruitment	171	Saliva/blood	Agilent SureSelect v4/5/6	Illumina HiSeq2000/2500
Bipolar disorder (BD)	Goes et al. Mol Psychiatry 2019	97	Blood	Whole genome sequencing	Illumina HiSeq2000
Schizoaffective disorder (SCZAD)	Fromer et al. Nature 2014	86	Blood	Agilent SureSelect v1/2<br>Nimblegene SeqCapEZ2	Illumina HiSeq2000/GAIIx
Autism spectrum disorder (ASD)	Krumm et al. Nat Genet. 2015	1646<br>in our study	Blood	Nimblegene SeqCapEZ1/2	Illumina HiSeq2000
Control (ASD sibling)	Krumm et al. Nat Genet. 2015	1646<br>in our study	Blood	Nimblegene SeqCapEZ1/2	Illumina HiSeq2000

Supplementary Table 3. Workflow of two pipelines for gDNM detection

Pipeline	Unified pipeline	Discovery pipeline
**Purpose**	case-control comparison	extensive variant discovery*
**Priority**	specificity	balancing sensitivity and specificity
**Samples**	257 trios with BD<br>1646 quartets with ASD	257 trios with BD
**Read QC**	FastQC-0.11.7	FastQC-0.11.7
**Reference genome**	GRCh37	hg38
**Alignment**	BWA-0.7.5a -M -k 17	BWA-0.7.17 -M -k 17
**Sort**	Picard-1.000 SortSam	Picard-2.18.3 SortSam
**De-duplication**	Picard-1.000 MarkDuplicates	Picard-2.18.3 MarkDuplicates
**Indel realignment**	GATK-2.7-4 RealignerTargetCreator/IndelRealigner<br>(family-based)<br>file: 1000G_phase1.indels.b37.vcf<br>file: Mills_and_1000G_gold_standard.indels.b37.vcf	NA
**BQSR**	GATK-2.7-4 BaseRecalibrator/ApplyRecalibration<br>file: dbsnp_137.b37.vcf<br>file: 1000G_phase1.indels.b37.vcf<br>file: Mills_and_1000G_gold_standard.indels.b37.vcf	GATK-4.0.12.0 BaseRecalibrator/ApplyRecalibration<br>file: dbsnp138.hg38.vcf<br>file: Homo_sapiens_assembly38.known_indels.vcf<br>file: Mills_and_1000G_gold_standard.indels.hg38.vcf
**Variant calling**	GATK-2.7-4 ReduceReads/HaplotypeCaller<br>target: Krumm interval (padding 20bp)<br>samples: joint call 20-26 fams	GATK-4.0.12.0 HaplotypeCaller -ERC GVCF<br>target: GENCODEv29+RefSeq (padding 50bp)<br>GATK-4.1.0.0 GenomicsDB/GenotypeGVCF
**VQSR**	GATK-2.7-4 VariantRecalibrator/ApplyRecalibration<br>(select PASS)<br>file: hapmap_3.3.b37.sites.vcf<br>file: 1000G_omni2.5.b37.sites.vcf<br>file: 1000G_phase1.snps.high_confidence.vcf<br>file: dbsnp_137.b37.vcf<br>file: Mills_and_1000G_gold_standard.indels.b37.vcf	GATK-4.1.0.0 VariantRecalibrator/ApplyRecalibration<br>(select PASS)<br>file: hapmap_3.3.hg38.vcf<br>file: 1000G_omni2.5.hg38.vcf<br>file: 1000G_phase1.snps.high_confidence.hg38.vcf<br>file: dbsnp138.hg38.vcf<br>file: Mills_and_1000G_gold_standard.indels.hg38.vcf
**Normalization**	bcftools-1.19 norm -m- -f GRCh37	bcftools-1.19 norm -m- -f hg38
**DNM call**	Triodenovo-0.06 (default parameters)	Triodenovo-0.06 (default parameters)
**filtering by a cohort of parents**	bcftools-1.19 filter: exclude AC=1 or more variants in parental cohort (around 250 fams in one batch)	bcftools-1.19 filter: exclude AC=2 or more variants in parental cohort (around 250 fams in one batch)
**DNMFilter**	DNMFilter-0.1.1: score ≥ 0.90 (SNV) or 0.95 (INDEL)	DNMFilter-0.1.1: score ≥ 0.85
**Manual inspection**	NA	IGV-2.5.2 exclude probable false calls:<br>(i) supported by less than two reads in IGV<br>(ii) coinciding with other two or more variant positions in the same read (suggestive of misalignment)<br>(iii) with two or more reads supporting the variant in the parent(s) (likely due to transmission or systematic errors)
**Liftover**	NA	Picard-2.18.3 LiftoverVcf from hg38 to GRCh37

BQSR: Base quality score recalibration, VQSR: Variant quality score recalibration. *Extensive variant discovery was performed by combining the results from the Unified and the Discovery pipelines.

Supplementary Table 4. Computational annotation for the detected variants

Item	Software/parameter	Source
Gene/Ensemble ID	SnpEff-4.3 GRCh37.75 -canon	http://snpeff.sourceforge.net/
SO (sequence ontology)	SnpEff-4.3 GRCh37.75 -canon	http://snpeff.sourceforge.net/
exon#/cDNA/HGSV.p	SnpEff-4.3 GRCh37.75 -canon	http://snpeff.sourceforge.net/
CADD	CADD GRCh37-v1.4 phred-scaled score	https://cadd.gs.washington.edu/
nonpsych.pLI	fordist_cleaned_nonpsych_z_pli_rec_null_data.txt	ftp://ftp.broadinstitute.org/pub/ExAC_release/release0.3/ functional_gene_constraint/
gnomAD.non_neuro.AF	gnomad.exomes.r2.1.1.sites.vcf.bgz (non_neuro_AF)	https://gnomad.broadinstitute.org/downloads
gnomAD.all.AF	gnomad.exomes.r2.1.1.sites.vcf.bgz (AF)	https://gnomad.broadinstitute.org/downloads
ToMMo.AF	ToMMo 3.5KJPNv2 Allele Frequency Panel (v20181105open; Unfiltered) AF	https://jmorp.megabank.tohoku.ac.jp/202001/downloads /legacy/#variant
TCGA576	> Exploration > Genes (576)	https://portal.gdc.cancer.gov/
OMIM	Table S1	Sanders et al. Nat Med. 2019
SIFT_pred	dbNSFP-4.0a SIFT_pred	https://sites.google.com/site/jpopgen/dbNSFP
Polyphen2_HDIV_pred	dbNSFP-4.0a Polyphen2_HDIV_pred	https://sites.google.com/site/jpopgen/dbNSFP
Polyphen2_HVAR_pred	dbNSFP-4.0a Polyphen2_HVAR_pred	https://sites.google.com/site/jpopgen/dbNSFP
LRT_pred	dbNSFP-4.0a LRT_pred	https://sites.google.com/site/jpopgen/dbNSFP
MutationTaster_pred	dbNSFP-4.0a MutationTaster_pred	https://sites.google.com/site/jpopgen/dbNSFP
MutationAssessor_pred	dbNSFP-4.0a MutationAssessor_pred	https://sites.google.com/site/jpopgen/dbNSFP
PROVEAN_pred	dbNSFP-4.0a PROVEAN_pred	https://sites.google.com/site/jpopgen/dbNSFP

Supplementary Table 5. Workflow for pzDNM detection

Process	Software/command
**Recalibrated bam**	from Discovery pipeline
**Reference genome**	hg38
**Panel of Normal (PoN)**	GATK-4.1.0.0 Mutect2/CreatePanelOfNormals<br>samples: cohort of parents with no BD, SCZAD nor SCZ
**pzDNM calling**	GATK-4.1.0.0 Mutect2<br>tumor: proband.bam<br>normal: mother.bam and father.bam<br>target: GENCODEv29+RefSeq (padding 50bp)<br>file: somatic-hg38_af-only-gnomad.hg38.vcf
**Contamination estimate**	GATK-4.1.0.0 GetPileupSummary/CalculateContamination<br>target: somatic-hg38_small_exac_common_3.hg38.vcf
**Software filtering**	GATK-4.1.0.0 FilterMutectCall (select PASS)
**CNV calling**	XHMM-1.0 exon-capturing-based calling<br>follow the tutorial (https://atgu.mgh.harvard.edu/xhmm/tutorial.shtml)<br>select Q_SOME ? 60 in any member in the family as CNV-called regions<br>exclude candidates hitting CNV-called regions
**Hard filtering**	bcftools-1.19 filter (include)<br>P-value binomal test of allele counts of alt to total in the proband < 0.01 (assuming null hypothesis of ref : alt = 1 : 1)<br>allele count of the proband ≥ 5<br>variant calls both on forward and reverse reads<br>the allele count of the parents ≥ 1<br>depth of parents ≥ 10<br>TLOD (fidelity score by GATK-4.1.0.0 MuTect2) ≥ 5
**Manual inspection**	IGV-2.5.2 exclude probable false calls:<br>(i) supported by less than two reads in IGV<br>(ii) coinciding with other two or more variant positions in the same read (suggestive of misalignment)<br>(iii) with two or more reads supporting the variant in the parent(s) (likely due to transmission or systematic errors)
**Liftover**	Picard-2.18.3 LiftoverVcf from hg38 to GRCh37

Supplementary Table 6. CHIP candidate genes in previous studies

Source	File	CHIP candidate genes
Genovese et al.<br>N Engl J Med. 2014	Table S3	*DNMT3A, SF3B1, MYD88, TET2, JAK2, ATM, CBL, IDH2, TP53, STAT3, PPM1D, SRSF2, ASXL1, U2AF1*
Jaiswal et al.<br>N Engl J Med. 2014	Table S3	*ASXL1, BCL11B, BCOR, BCORL1, BIRC3, BRAF, BRCC3, CARD11, CBL, CD58, CD79B, CNOT3, CREBBP, CUX1, DDX3X, DNMT3A, EP300, ETV6, EZH2, FAM46C, FBXW7, FLT3, FOXP1, GNAS, GNB1, HIST1H1C, IDH2, IKZF1, JAK2, JAK3, JARID2, KDM6A, KIT, KLHL6, KRAS, LUC7L2, MLL2, MPL, MYD88, NOTCH1, NOTCH2, NRAS, PDSS2, PHF6, PIK3CA, PRDM1, PRPF40B, PTPN11, RAD21, RIT1, RPS15, SETD2, SETDB1, SF1, SF3A1, SF3B1, SFRS2, SMC1A, SMC3, STAG1, STAG2, STAT3, SUZ12, TBL1XR1, TET1, TET2, TNFAIP3, TNFRSF14, TP53, U2AF1, ZRSR2*
Jaiswal et al.<br>N Engl J Med. 2014	Table S5	*APC, CASP8, GPS2, MAP3K1, NFE2L2, PIK3R1, VHL*
Xie et al.<br>Nat Med. 2014	Figure 5	*ASXL1, CEBPA, DNMT3A, FLT3, GNAS, IDH1, JAK2, NPM1, NRAS, PHF6, PPM1D, RUNX1, SF3B1, SH2B3, STAG2, TET2, U2AF1, WT1*
Desai et al.<br>Nat Med. 2018	Supplementary Dataset	*ASXL1, ASXL2, ATRX, BCOR, BRAF, CALR, CARD11, CBL, CBLB, CEBPA, CREBBP, CUX1, DNMT3A, ETV6, EZH2, FAM46C, FLT1, FLT3, GATA1, GATA2, GNAS, HRAS, IDH1, IDH2, IKZF1, JAK1, JAK2, KDM6A, KIT, KRAS, NOTCH1, NPM1, NRAS, PAX5, PHF6, RAD21, RUNX1, SETBP1, SF3B1, SRSF2, TET1, TET2, TP53, U2AF1, WT1*
Abelson et al.<br>Nature 2018	Table S2.1	*ASXL1, BCOR, CALR, CBL, DNMT3A, FLT3, GATA2, IDH1, IDH2, JAK2, KDM6A, KIT, KMT2C, KRAS, NF1, NRAS, PHF6, PTPN11, RUNX1, SF3B1, SRSF2, TET2, TP53, U2AF1*
Abelson et al.<br>Nature 2018	Table S2.2	*ASXL1, BCOR, CBL, CEBPA, DNMT3A, IDH1, IDH2, JAK2, KMT2C, KMT2D, KRAS, NF1, NRAS, PTPN11, RAD21, RUNX1, SF3B1, SRSF2, TET2, TP53, U2AF1*
LeeSix et al.<br>Nature 2018	Table S3	*ASXL1, BCORL1, BRAF, CARD11, CBL, CD58, CD79B, CNOT3, CREBBP, DNMT3A, EP300, EZH2, FBXW7, FLT3, GNAS, GNB1, IDH1, IDH2, JAK2, JAK3, KIT, KRAS, MPL, MYD88, NPM1, NRAS, PHF6, PIK3CA, PPM1D, PTPN11, RAD21, RPS15, SF3A1, SF3B1, SRSF2, STAG1, STAG2, STAT3, TET2, TP53, U2AF1*

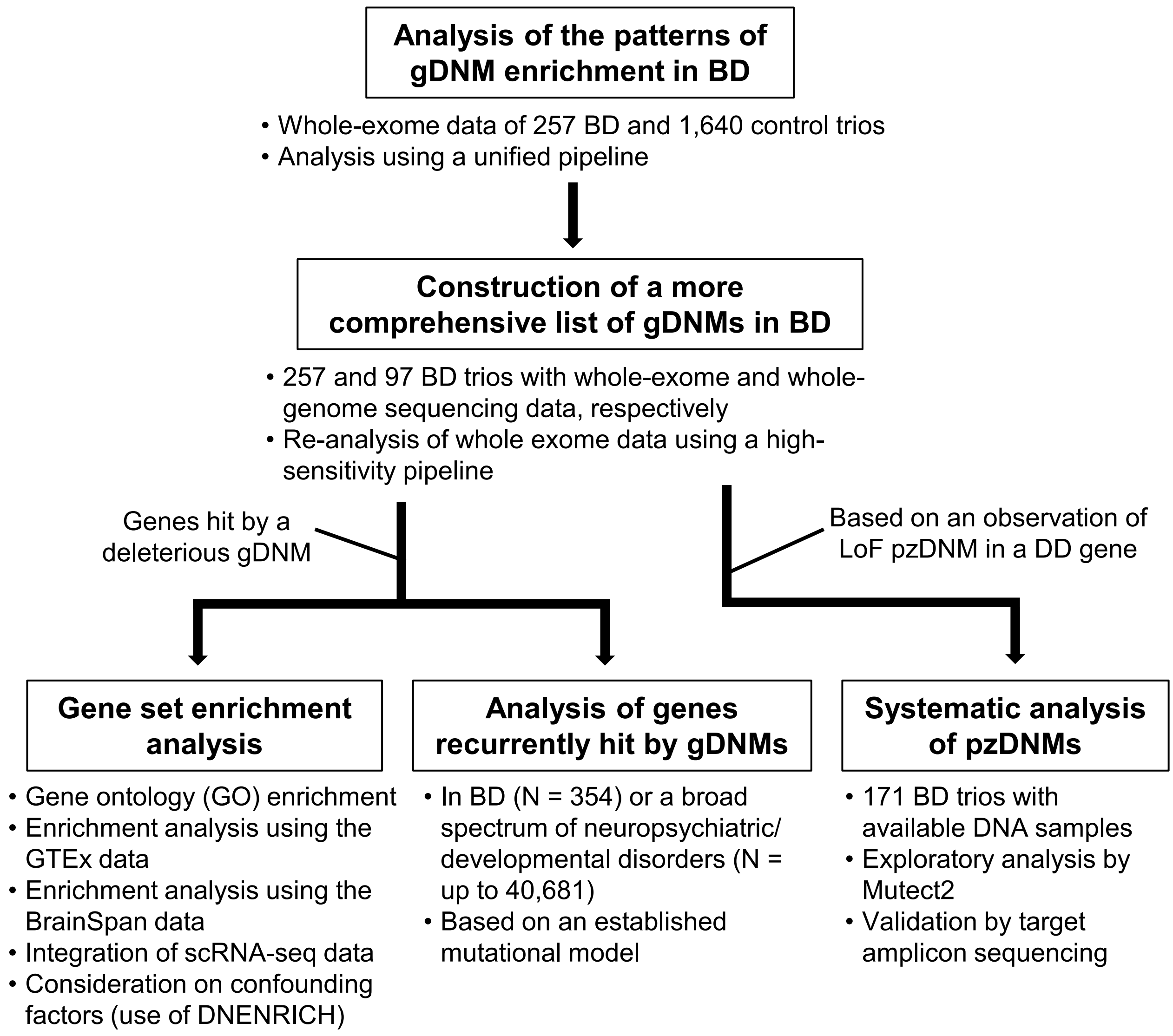

Supplementary Figure 1. An overview of the study design

Brief description of the resources and methods used for each analysis are shown as a bulleted list. BD: bipolar disorder, DD: developmental disorder, gDNM: germline *de novo* mutation, GTEx: Genotype-Tissue Expression, LoF: loss-of-function, pzDNM: postzygotic *de novo* mutation.

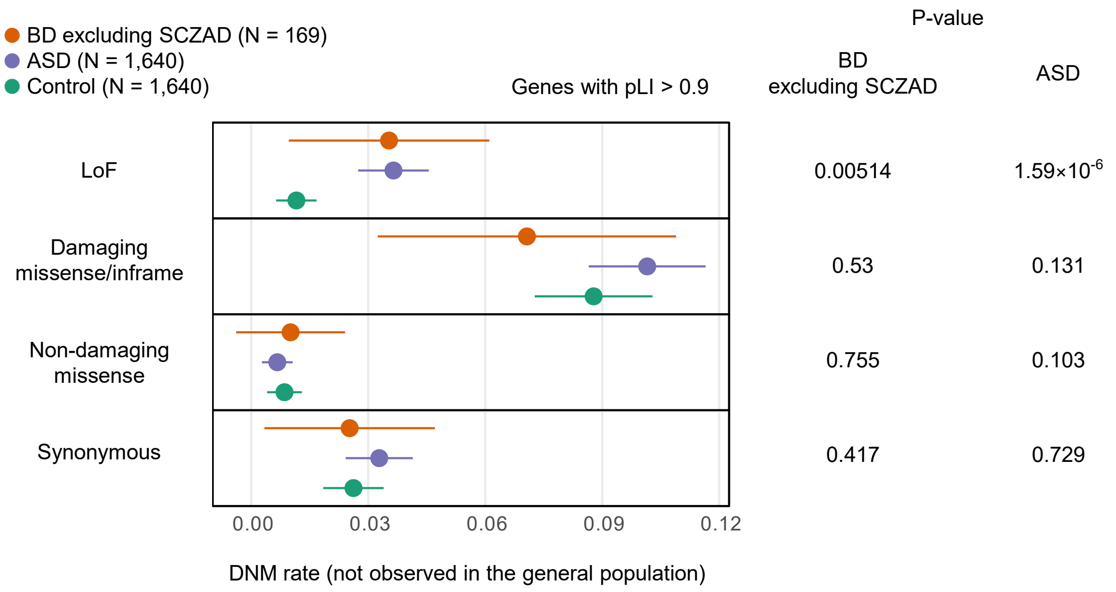

Supplementary Figure 2. Enrichment of LoF gDNMs hitting constrained genes in the group of BD excluding SCZAD

Plots of per-individual rates of gDNMs not observed in the general population and hitting a constrained gene in the groups of BD excluding SCZAD (orange, N = 169), ASD (purple, N = 1,640), and controls (green, N = 1,640) are shown. The gDNMs are classified into the following four types: loss-of-function (LoF), damaging missense/inframe indel, non-damaging missense, and synonymous. Damaging missense gDNMs are defined as those with a Combined Annotation Dependent Depletion (CADD) score > 15. Error bars indicate 95% confidence intervals. P-values calculated by one-tailed permutation tests comparing gDNM rates in BD or ASD to controls are shown on the right of the plots. The mean of gDNM counts in the affected and unaffected groups for each mutational type is indicated as the colored point accompanied by the error bars (95% confidence intervals).

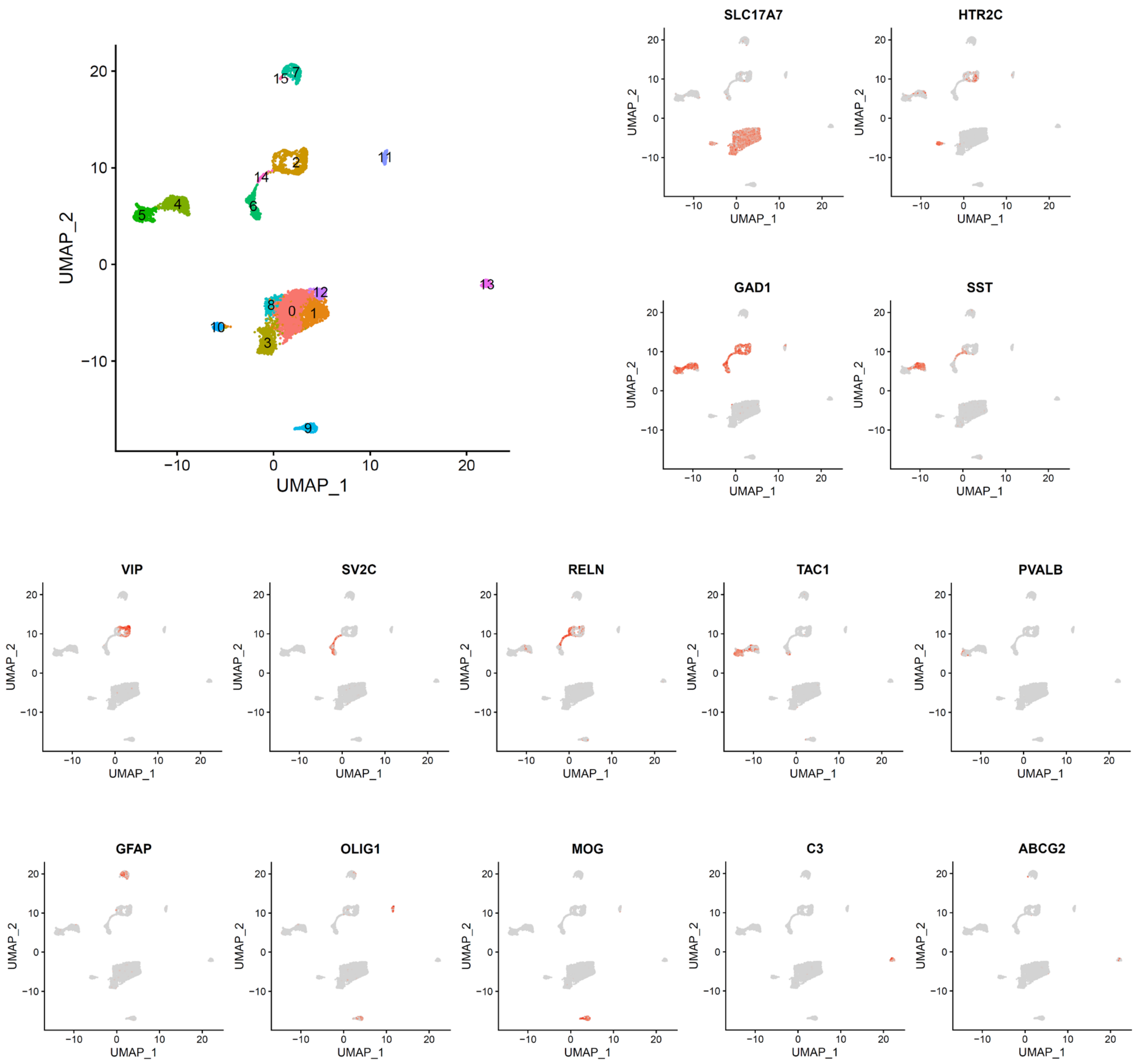

Supplementary Figure 3. Detailed information on marker gene expression in cell cluster analysis

UMAP representation of 16 cell clusters (c0 - 15) identified from single-nucleus RNA sequencing data of human adult anterior cingulate cortices is displayed in the top-left panel. The other panels show expression patterns of each marker genes (the red dots indicate cells with high expression of the corresponding marker gene). A general excitatory neuron marker *SLC17A7* was expressed in the clusters 0, 1, 3, 8, 10, and 12. Of these excitatory neuron clusters, the cluster 10 cells specifically expressed *HTR2C*. A general interneuron marker *GAD1* was expressed in the clusters 2, 4, 5, 6, and 14. The clusters 2 and 4 were characterized with expression of *VIP* and *SST*, respectively. *SV2C* was expressed in the clusters 6 and 14, whereas expression of *RELN* was negative in the cluster 14. The cluster 5 was characterized with expression of *TAC1*. Also, *PVALB* expression was specific to the cluster 5. The other clusters were characterized by expression of *GFAP* (astrocyte), *OLIG1* (oligodendrocyte), *MOG* (matured oligodendrocyte), *C3* (microglia), and *ABCG2* (endothelial cell). Based on the expression patterns of these genes, we annotated each cluster as shown in **Fig. 3a** in the main text.

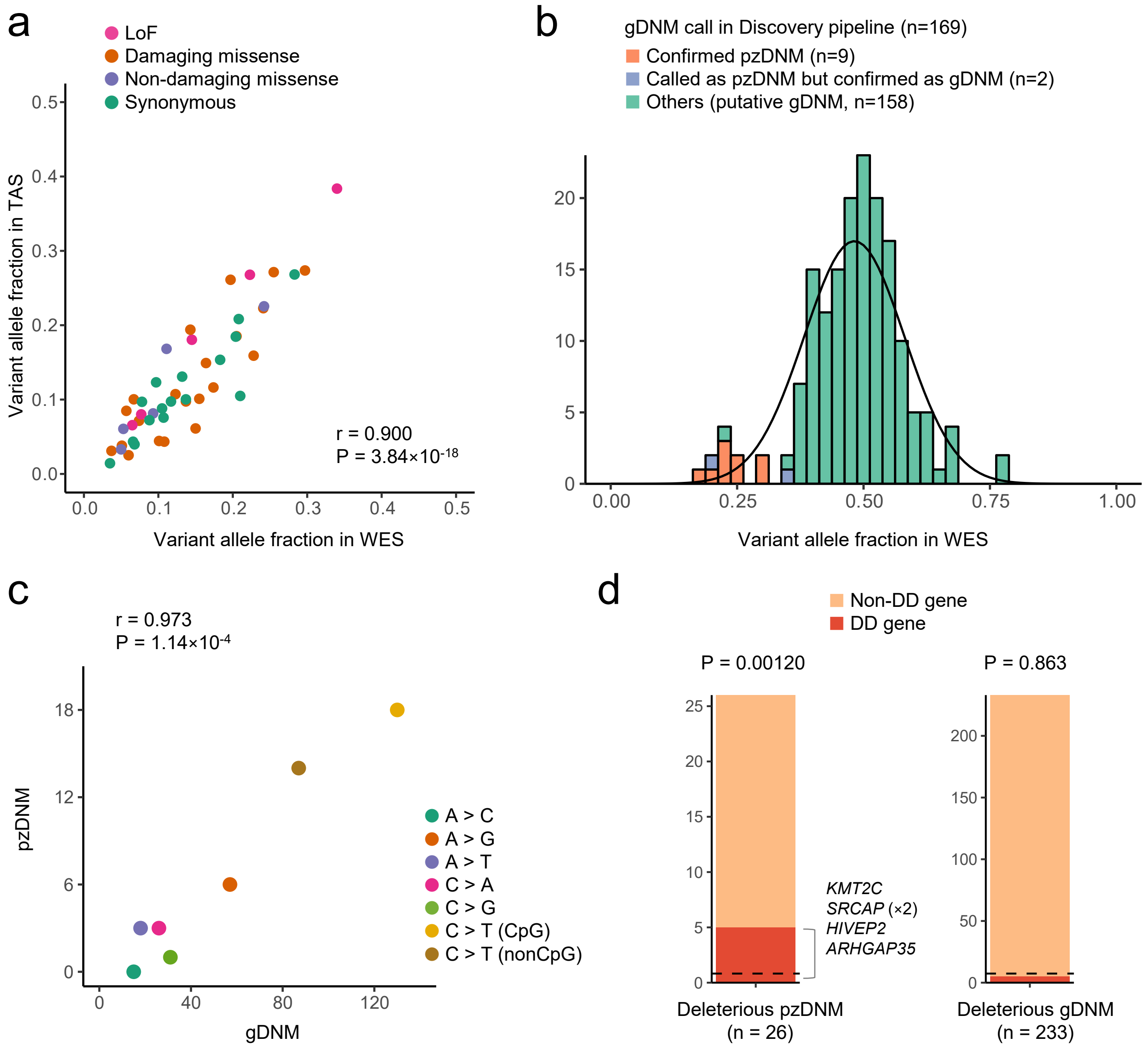

Supplementary Figure 4. Properties of the validated pzDNMs in BD

(a) Plots of the variant allele fractions (VAFs) in whole-exome sequencing (WES, x-axis) and target amplicon sequencing (TAS, y-axis) for 47 validated pzDNM. Four functional types of variants are color-coded as described above the plot. Pearson's correlation between VAFs in WES and TAS and the corresponding uncorrected p-value (one-tailed) are shown on the right bottom. **(b)** A histogram of VAFs of the gDNM calls in the Discovery pipeline (n = 169). Of these, eleven were also called by MuTect2 for pzDNM detection. Nine of the eleven calls (orange bars in the histogram) were validated as true pzDNMs by TAS, while the remaining two calls (purple) were validated as gDNMs. The others (n = 158, green) were not called by MuTect2 and are considered as true gDNMs in this study. The simulated normal distribution for all the gDNMs is shown as the black curve. No significant outlier variant was detected (Grubbs test P = 0.216). **(c)** Plots of the numbers of the seven substitution patterns of single nucleotide gDNMs (x-axis, n = 364) and pzDNMs (y-axis, n = 45). The substitution patterns are color-coded as shown on the right. There is a strong correlation between VAFs of gDNM and pzDNM (Pearson's r and the corresponding uncorrected p-value [one-tailed] are shown on the top left). The distributions of the substitution patterns are not significantly different between pzDNMs and gDNMs (P = 0.546, Fisher's exact test for a 7×2 contingency table). **(d)** Proportions of the deleterious (LoF and damaging missense) pzDNMs and gDNMs hitting a known DD gene in BD. The DNMs here include those found in the general population. The dotted lines indicate the theoretical expectation based on an established mutational model by Samocha et al. The p-values calculated by a comparison between the observation and the expectation (one-tailed binomial test) are shown above the bars.

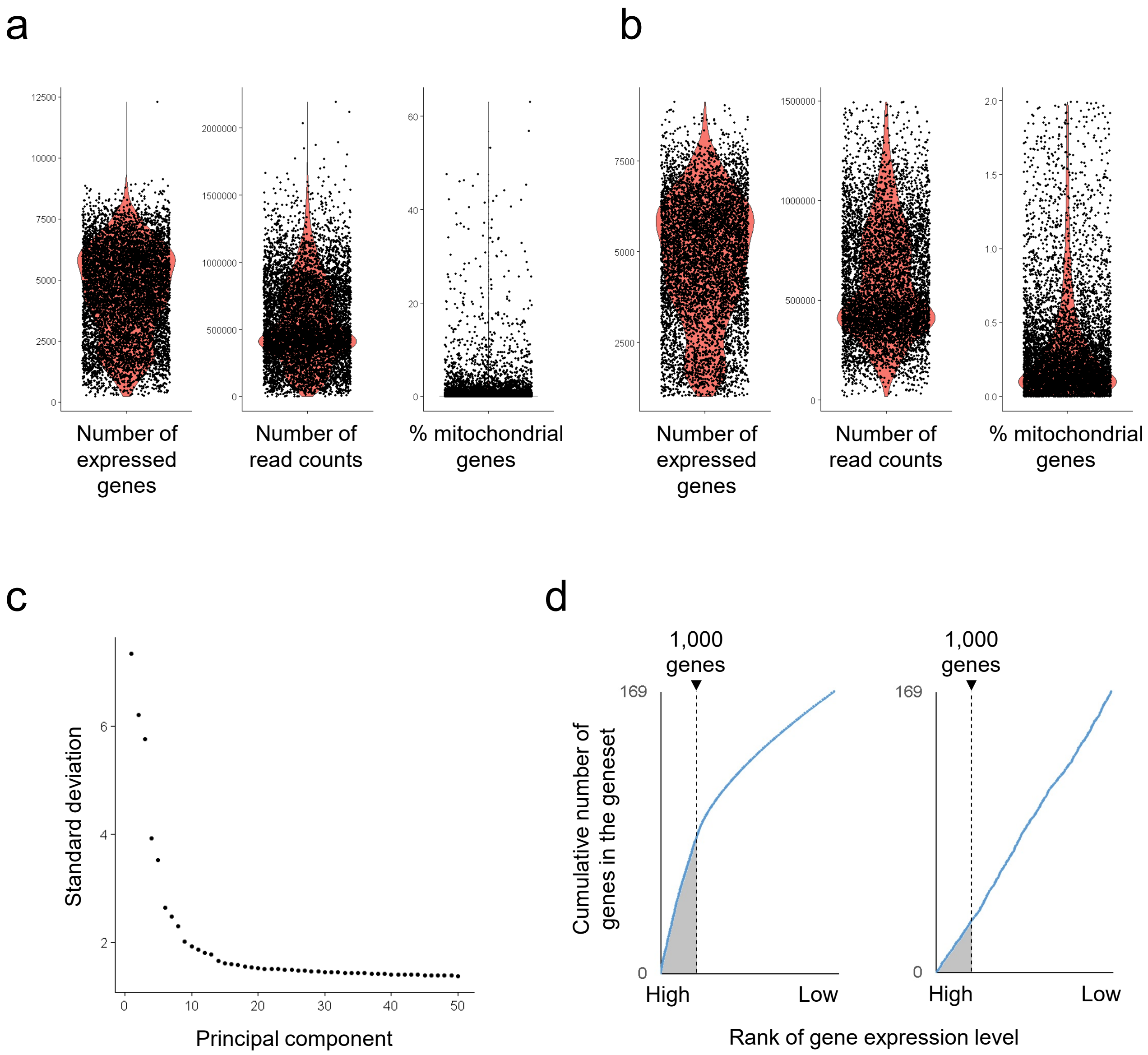

Supplementary Figure 5. Preprocessing for the single-cell (nucleus) enrichment analysis of the genes hit by deleterious gDNMs in BD

(a) and **(b)** Violin plots of the number of expressed genes, the number of unique read counts, and the proportion of the reads mapped onto the mitochondrial genome per cell before **(a)** and after **(b)** filtering. **(c)** An elbow plot of the first 50 principal components (PCs). We used the first 20 PCs for the downstream analysis. **(d)** Schematic representation of the area under the curve (AUC) calculation for each cell. The gray shaded areas indicate AUC. We used the information of the most highly expressed 1,000 genes to avoid potential bias due to the number of expressed genes in each cell. Left: an example of a cell in which the genes hit by deleterious gDNMs in BD are preferentially expressed. Right: an example of a cell in which ranks of the genes hit by deleterious gDNMs in BD are randomly distributed.
