# Supplementary material for: Systematic analysis of exonic germline and postzygotic de novo mutations in bipolar disorder
Source: Nat Commun. 2021 Jun 18;12:3750. doi: 10.1038/s41467-021-23453-w (PMC8213845; doi:10.1038/s41467-021-23453-w)
Supplement: Supplementary file 3 — Description of Additional Supplementary Files [file 41467_2021_23453_MOESM3_ESM.pdf]

Description of Additional Supplementary Files

File Name: Supplementary Data 1
Description: De novo mutations detected as gDNMs from 354 BD trios

File Name: Supplementary Data 2
Description: Gene ontology enrichment analysis of genes hit by deleterious gDNMs in BD

File Name: Supplementary Data 3
Description: Genes upregulated in the cluster 8 cells (c8 sigunature genes)

File Name: Supplementary Data 4
Description: Gene ontology enrichment analysis of c8 signature genes

File Name: Supplementary Data 5
Description: Genesets used in the characterization of c8 signature genes

File Name: Supplementary Data 6
Description: Validated postzygotic de novo mutations (pzDNMs) in 171 trios with bipolar disorder

File Name: Supplementary Data 7
Description: Phenotypic and alignment information of 256 trios with BD in our analysis

File Name: Supplementary Data 8
Description: Primer sequences in TAS validation experiments
