# Supplementary material for: Systematic analysis of exonic germline and postzygotic de novo mutations in bipolar disorder
Source: Nat Commun. 2021 Jun 18;12:3750. doi: 10.1038/s41467-021-23453-w (PMC8213845; doi:10.1038/s41467-021-23453-w)
Supplement: Supplementary file 12 — Reporting Summary [file 41467_2021_23453_MOESM12_ESM.pdf]

nature research

Corresponding author(s): Atsushi Takata, Tadafumi Kato

Last updated by author(s): Apr 14, 2021

Reporting Summary

Nature Research wishes to improve the reproducibility of the work that we publish. This form provides structure for consistency and transparency in reporting. For further information on Nature Research policies, see our Editorial Policies and the Editorial Policy Checklist.

Statistics

For all statistical analyses, confirm that the following items are present in the figure legend, table legend, main text, or Methods section.

n/a	Confirmed	
☐	☒	The exact sample size (*n*) for each experimental group/condition, given as a discrete number and unit of measurement
☐	☒	A statement on whether measurements were taken from distinct samples or whether the same sample was measured repeatedly
☐	☒	The statistical test(s) used AND whether they are one- or two-sided<br>*Only common tests should be described solely by name; describe more complex techniques in the Methods section.*
☐	☒	A description of all covariates tested
☐	☒	A description of any assumptions or corrections, such as tests of normality and adjustment for multiple comparisons
☐	☒	A full description of the statistical parameters including central tendency (e.g. means) or other basic estimates (e.g. regression coefficient) AND variation (e.g. standard deviation) or associated estimates of uncertainty (e.g. confidence intervals)
☐	☒	For null hypothesis testing, the test statistic (e.g. *F*, *t*, *r*) with confidence intervals, effect sizes, degrees of freedom and *P* value noted<br>*Give P values as exact values whenever suitable.*
☒	☐	For Bayesian analysis, information on the choice of priors and Markov chain Monte Carlo settings
☐	☒	For hierarchical and complex designs, identification of the appropriate level for tests and full reporting of outcomes
☐	☒	Estimates of effect sizes (e.g. Cohen's *d*, Pearson's *r*), indicating how they were calculated

Our web collection on statistics for biologists contains articles on many of the points above.

Software and code

Policy information about availability of computer code

Data collection	Raw sequencing data were produced on Illumina HiSeq 2000/2500. Sequencing reads were processed according to the GATK best practice workflows. The full pipelines including software version and parameters are fully described in Supplementary Table S9 and S12.
Data analysis	Variant annotations are described in Supplementary Table S11. Gene set enrichment analyses were performed by DNENRICH (no version information, Fromer et al. Nature 2014). Network visualization was conducted by EnrichmentMap v3.2.1 plugin of Cytoscape v3.7.2. Single nucleus RNA-seq analyses were performed by AUCell v1.8.0 and Seurat v3.1.2. Statistical analyses were conducted in R (v3.4.2) using standard R packages for each statistical test as follows: perm (permutation test), poisson.test (poisson test), binom.test (binomial test), phyper (hypergeometric test), wilcox.exact (exact Wilcoxon rank sum test), cor.test (Pearson's correlation), grubbs.test (Grubbs test), fisher.test (Fisher's exact test), and p.adjust (multiple testing collection). The custom code for outlier detection is attached as

For manuscripts utilizing custom algorithms or software that are central to the research but not yet described in published literature, software must be made available to editors and reviewers. We strongly encourage code deposition in a community repository (e.g. GitHub). See the Nature Research guidelines for submitting code & software for further information.

Data

Policy information about availability of data

All manuscripts must include a data availability statement. This statement should provide the following information, where applicable:
- Accession codes, unique identifiers, or web links for publicly available datasets
- A list of figures that have associated raw data
- A description of any restrictions on data availability

De novo mutations discovered from BD trios are listed in Supplementary Table S1 and S7. The sequence data of study participants who provided informed consent for database registration (N of trios = 144) are available through the National Bioscience Database Center (NBDC) Human Database, Japan, with accession code

JGAS000273/JGAD000379. The sequence data from the trios with no consent for database registration (N of trios = 27) can be accessed via formal collaboration due to the contents of the obtained informed consent. This study's key resources are summarized with source information in Supplementary Table 6, 8, and 11. We used the following data from the public database: Exome data from trios with schizoaffective disorder (NCBI dbGaP phs000687.v1.p1 with authorization) [https://www.ncbi.nlm.nih.gov/gap/], Exome data from quartets with autism spectrum disorder (SFARI SSC WES3 with authorization) [https://www.sfari.org/resource/sfari-base/], De novo mutations in bipolar disorder [https://doi.org/10.1038/s41380-019-0611-1], GRCh37 human reference genome [ftp://gsapubftp-anonymous@ftp.broadinstitute.org/bundle/b37/], GRCh37 variant information files [ftp://gsapubftp-anonymous@ftp.broadinstitute.org/bundle/b37/], hg38 human reference genome [https://console.cloud.google.com/storage/browser/genomics-public-data/resources/broad/hg38/v0], hg38 variant information files [https://console.cloud.google.com/storage/browser/genomics-public-data/resources/broad/hg38/v0], ExAC 0.3 nonpsychiatric pLI [ftp://ftp.broadinstitute.org/pub/ExAC_release/release0.3/functional_gene_constraint/], gnomAD r2.1.1 [https://gnomad.broadinstitute.org/downloads], ToMMo 3.5JPN [https://jmorp.megabank.tohoku.ac.jp/202001/downloads/legacy/#variant].

Field-specific reporting

Please select the one below that is the best fit for your research. If you are not sure, read the appropriate sections before making your selection.

☒ Life sciences ☐ Behavioural & social sciences ☐ Ecological, evolutionary & environmental sciences

For a reference copy of the document with all sections, see nature.com/documents/nr-reporting-summary-flat.pdf

Life sciences study design

All studies must disclose on these points even when the disclosure is negative.

Sample size	No statistical methods were used to pre-determine sample sizes. We obtained data from BD trios as much as possible.<br>513 samples from 171 parent-child trios with BD were exome-sequenced and passed QC. This sample size is the largest among the published studies for de novo short variants in BD. The exome sequencing data of 86 parent-child trios with schizoaffective disorder were obtained from dbGaP after the authorization process. The exome sequencing data of 1,772 families with ASD were obtained from SFARI base after receiving an approval. The list of de novo mutations from whole genome sequencing in 97 BD trios was obtained from Goes et al. Molecular Psychiatry 2019.
Data exclusions	No data from BD samples were excluded after QC. In the comparison of per-individual rates of de novo mutations between BD, ASD, and Control (ASD sibling) demonstrated in Figure 1 and Supplementary Figure 2, 132 quartets with ASD and Control were excluded from the analysis due to the following reasons: no variant quality score recalibration (VQSR), low qualities after VQSR, inadequately formatted files, suspected parent-child relationship, detection of outliers (described in the Methods section).
Replication	Representative germline de novo mutations were validated by Sanger sequencing. The mutations confirmed by Sanger sequencing are denoted in Supplementary Table 1 (n = 103). One germline de novo mutation in ARID1B was validated by target amplicon sequencing because of long deletion. The remaining mutations were not validated by Sanger sequencing. Still, the validation rate was high enough (96.8% for Unified and 93.4% for Discovery pipeline) to proceed to the systematic analysis of de novo mutations. All the postzygotic de novo mutations listed in our study were validated by targeted amplicon sequencing (n = 47, Supplementary Table 7).
Randomization	This study does not require randomization into the groups because this study is not an experimental study. The sex of the proband is a potential confounding factor in the comparison of per-individual rates of germline de novo mutations (gDNMs) between BD, ASD, and Control (ASD sibling). We excluded de novo mutations on sex chromosomes in this comparison (Krumm pipeline, see Methods). The proband age is not regarded as a possible confounding factor for gDNM analysis but is considered a confounding factor for pzDNM analysis. We refrained from comparing pzDNM rates between BD and Control in this study.
Blinding	This study does not require blinding to group allocation during data collection or analysis, because this study is not an experimental study. We analyzed the affected and unaffected groups with the same procedures.

Reporting for specific materials, systems and methods

We require information from authors about some types of materials, experimental systems and methods used in many studies. Here, indicate whether each material, system or method listed is relevant to your study. If you are not sure if a list item applies to your research, read the appropriate section before selecting a response.

Materials & experimental systems

n/a	Involved in the study	
☒	☐	Antibodies
☒	☐	Eukaryotic cell lines
☒	☐	Palaeontology and archaeology
☒	☐	Animals and other organisms
☐	☒	Human research participants
☒	☐	Clinical data
☒	☐	Dual use research of concern

Methods

n/a	Involved in the study	
☒	☐	ChIP-seq
☒	☐	Flow cytometry
☒	☐	MRI-based neuroimaging

Human research participants

Policy information about studies involving human research participants

Population characteristics	BD probands in our own recruitment (N = 171)<br>Mean age of the probands at recruitment: 35.9 years old<br>Male probands: 74 individuals<br>Female probands: 97 individuals<br>Continental genotypic ancestry: Japan (East Asia)<br>Current diagnosis: Bipolar disorder or schizoaffective disorder
Recruitment	One hundred seventy-one probands with BD and their parents were recruited through Bipolar Disorder Research Network Japan (BDRNJ, http://bipolar.umin.jp/), university hospitals, and primary care clinics across Japan. Participants recruited through BDRNJ are possibly biased to the individuals interested in scientific research. Although we did not assess the participants' cognitive abilities in this study, the participants from BDRNJ can have higher cognitive abilities than the individuals with bipolar disorder in general.
Ethics oversight	This study was designed according to the Helsinki declaration and approved by RIKEN Wako Research Ethics First Committee, The Ethical Review Board of Juntendo University Faculty of Medicine, Yokohama City University Human Genome and Gene Research Ethics Committee, Ethical Committee of Saitama Medical University, and the Ethical Review Boards for Human Genome Studies at Fujita Health University. RIKEN served as the headquarter for data collection and research diagnosis.

Note that full information on the approval of the study protocol must also be provided in the manuscript.
